# Relationship Between One-Leg Standing and Foot Sole Sensation Following Short-Term Immobilization of a Unilateral Lower Extremity

**DOI:** 10.7759/cureus.76031

**Published:** 2024-12-19

**Authors:** Takuro Ikeda, Shinichiro Oka, Naoki Nomura, Akari Suzuki, Kensuke Matsuda

**Affiliations:** 1 Department of Physical Therapy, School of Health Sciences, International University of Health and Welfare, Fukuoka, JPN; 2 Department of Physical Therapy, Faculty of Rehabilitation, Reiwa Health Sciences University, Fukuoka, JPN; 3 Department of Rehabilitation, Akiyama Clinic, Fukuoka, JPN

**Keywords:** cast immobilization, foot sole sensation, physical inactivity, postural control, young adults

## Abstract

Background: Several studies have suggested that approximately 10 hours of inactivity can reduce motor performance. Specifically, restricted lower limb movement may impair postural stability, subsequently increasing the incidence of falls. However, the relationship between postural sway and its related factors remains unclear. This study investigated the relationship between postural sway in the upright standing position and foot sole sensitivity after short-term immobilization.

Methods: Healthy young adults were enrolled. Each participant’s lower limb was immobilized for 10 hours using a soft bandage and a medical splint made from metal and soft urethane. The sway in the center of the pressure trajectory was measured before and after immobilization. Evaluation parameters included path length, mean velocity, and sway area. Foot sole sensitivity was assessed using an esthesiometer to measure two-point discrimination before cast application and immediately after cast removal.

Results: After cast removal, total and anterior-posterior path lengths, mean velocity and sway area increased, whereas big toe sensitivity decreased. However, no significant correlations were observed among these factors.

Conclusions: Our results suggest that short-term movement restriction induces acute changes in center of pressure (COP) movement and foot sensitivity. However, the COP movement was not associated with foot sensation, indicating that another factor may contribute to postural sway after cast removal.

## Introduction

Globally, many surgeons choose cast fixation as the preferred treatment for post-fracture patients to promote tissue repair and minimize bone dislocation. However, long-term movement restriction causes secondary functional disorders such as muscle atrophy [1]. Lack of movement due to prolonged bed rest causes loss of balance and increases the incidence of falls [2]. Similarly, cast immobilization procedures for treating fractures have been shown to impair balance during rehabilitation [3,4]. Moreover, movement restriction in the lower limbs may affect balance and increase the risk of falls.

Previous studies indicated that only a few hours of movement restriction can impair motor performance. For example, after 24 hours of cast immobilization, pointing tasks on a personal computer showed a delayed movement time [5]. Similarly, grasp-reaching tasks required a longer time to reach objects after 10 hours of cast application [6]. Immobilization of the upper extremity for 12 hours increased the hand-path area amplitude and showed a deviating trajectory of inter-joint coordination compared to that before cast application [7]. These results suggest that even brief periods of movement restriction can affect the upper limb movement accuracy, velocity, and motor control. A recent study on lower limb movement restriction found that after 10 hours, postural sway during a static upright stance increased after removal of a cast on one side compared with postural sway before cast application [8,9]. Thus, disuse of the lower limb, regardless of the presence or absence of a fracture or pain, may impair balance in the standing position, even in the early stages of immobilization. However, the relationship between postural sway and its related factors remains poorly understood.

Several brain studies have reported that motor restriction reduces cortical thickness and response in the cortical somatosensory cortex [10,11]. Plastic changes in the sensory nervous system following motor restriction may interfere with postural control. When standing on a foam surface [12] and after injecting a local anesthetic into the sole [13], the reliability of somatosensory information is reduced, and postural instability occurs. The sole of a human foot is only in contact with the ground in an upright position. Thus, inaccurate detection of somatosensory information from the sole can lead to reduced stability. Assuming that plasticity changes in the somatosensory nervous system are induced after cast removal, reduced foot sole sensitivity may inhibit the feedback and feedforward processes necessary for postural control [14]. However, whether foot sole sensation affects postural control in the upright position after cast removal remains unclear.

This study aimed to investigate the changes in and relationship between upright standing postural sway and foot sensation after short-term unilateral lower limb immobilization. We hypothesized that postural sway and decreased sensitivity would be greater after movement restriction and would be correlated. By establishing these relationships, the study may provide insight into factors contributing to postural sway following unilateral lower limb immobilization. These findings could inform future research on postural dysregulation after conservative treatment, such as cast immobilization, following trauma to the lower extremity.

## Materials and methods

Ethics statement

Written informed consent was obtained from all participants. The Ethics Committee of the International University of Health and Welfare approved this study on March 27, 2018 (17-Ifh-73), which conformed to the principles of the Declaration of Helsinki. The study was registered at the University Hospital Medical Information Network Clinical Trials Registry (UMIN-CTR) as a clinical trial on August 25, 2022 (Unique ID: UMIN000048744).

Participants

This observational study was conducted at the International University of Health and Welfare in Japan. Data were collected from June to September 2017. Twelve healthy male participants (age, 21.0 ± 0.7 years) were enrolled in this study. The inclusion criteria were as follows: (a) healthy young adults, (b) no prior participation in movement restriction studies, and (c) avoidance of strenuous activity and alcohol consumption for 24 hours prior to the experiment. The exclusion criteria were as follows: (a) history of injuries or diseases that influence balance and foot sensory function, (b) intake of medications that affect postural control, and (c) current involvement in physical training. All participants had normal or corrected-to-normal visual acuity.

Immobilization conditions

The non-dominant legs of the participants were immobilized from the lower one-third of the thigh to the proximal phalanx using a soft bandage and a medical splint (Soflatsine II; Taketora Corp., Yokohama, Japan). The question "Which leg do you choose to kick a ball?" allowed us to select the opposite leg (non-dominant leg) that performs the stabilizing role. Participants did not move their immobilized lower limbs for 10 hours (09:00 to 19:00); however, they were permitted to use crutches for daily activities during the immobilization period. The non-immobilized leg was equipped with thick-soled shoes to prevent the immobile side from supporting the weight when walking with crutches. During immobilization, participants were monitored hourly for signs of numbness or leg pain. At the end of the immobilization period, the researcher promptly removed the bandage and splint. However, participants were not allowed to move their immobilized legs until the end of the experiment.

Examination of standing postural stability

Postural sway was assessed based on the sway path of the center of pressure (COP). The COP was measured using a single force plate (Twin Gravicorder G-6100; Anima Corp., Tokyo, Japan) with a sampling frequency of 20 Hz and was stored on a personal computer for subsequent analysis. This frequency aligns with the Japanese Society for Equilibrium Research standards for clinical COP sway testing. Participants were instructed to remain standing on the non-dominant leg on the force plate barefoot, with the opposite knee slightly flexed, arms resting vertically, and eyes open. They focused on a visual target placed 2 m away at a 1° visual angle. Movement and speech were prohibited during the test. The test started 5 seconds after the participants stood on a single force plate to eliminate the influence of outliers. The recording time was 30 seconds, starting after the participant’s posture had stabilized. The COP was measured twice before and after the 10-hour immobilization period, and the average values were adopted. The total, anterior-posterior (A-P), and medial-lateral (M-L) trajectory lengths (cm), mean velocity (cm/s), and sway area (cm^2^) were selected as evaluation parameters. The trajectory length represented the distance traveled within the two-dimensional axes of the M-L and A-P planes observed within 30 seconds, and the sum of the two was calculated as the total path length (TPL). The A-P and M-L path lengths (PL_AP_ and PL_ML_, respectively) represent the motions on the A-P and M-L coordinate axes. The mean velocity, which was the average speed at which the COP traveled, was calculated by dividing the total distance by the trial duration. The sway area was calculated as the area bound by the outermost points of the TPL.

Examination of foot sensation

Foot sensation was assessed using an esthesiometer (Puranogisu pocket 100 mm, Shinwa, Japan) to evaluate two-point discrimination (TPD) at the sole before cast application and immediately after cast removal. This test was performed immediately before the examination of standing postural stability. Participants were placed on the examination table in the supine position with the knee extended, and measurements were taken at the big toe (BT), midfoot (MF), and heel foot (HF) of the fixed limb. During the examination, participants were instructed to relax their entire body and close their eyes. The researcher applied pressure to two adjacent points perpendicular to the long axis of the sole using the esthesiometer. For each site, the distance between two points on the esthesiometer was gradually reduced until the participant could no longer distinguish between them. During the test, when the participant described the two-point touch as "once" three times, the distance read by the esthesiometer was recorded as the TPD distance threshold in millimeters.

Data analysis

Statistical analyses were performed using Statistical Package for the Social Sciences (IBM SPSS Statistics for Windows, IBM Corp., Version 26.0, Armonk, NY) and R software (version 4.2.0; The R Foundation for Statistical Computing, Vienna, Austria). First, the normality of the dependent variable was confirmed using the Shapiro-Wilk test, and Student’s paired t-test was used to compare the conditions before cast application and immediately after cast removal. For significant differences within the group, repeated measures correlation (RMC) analysis was used to examine correlations between parameters before and after the 10-hour immobilization period [15]. The alpha value was a priori set at p < 0.05 for all analyses. Effect sizes were examined using Cohen’s d, where values of 0.2-0.4, 0.4-08, and >0.8 indicated small, medium, and large effects, respectively.

## Results

**Figure 1:**
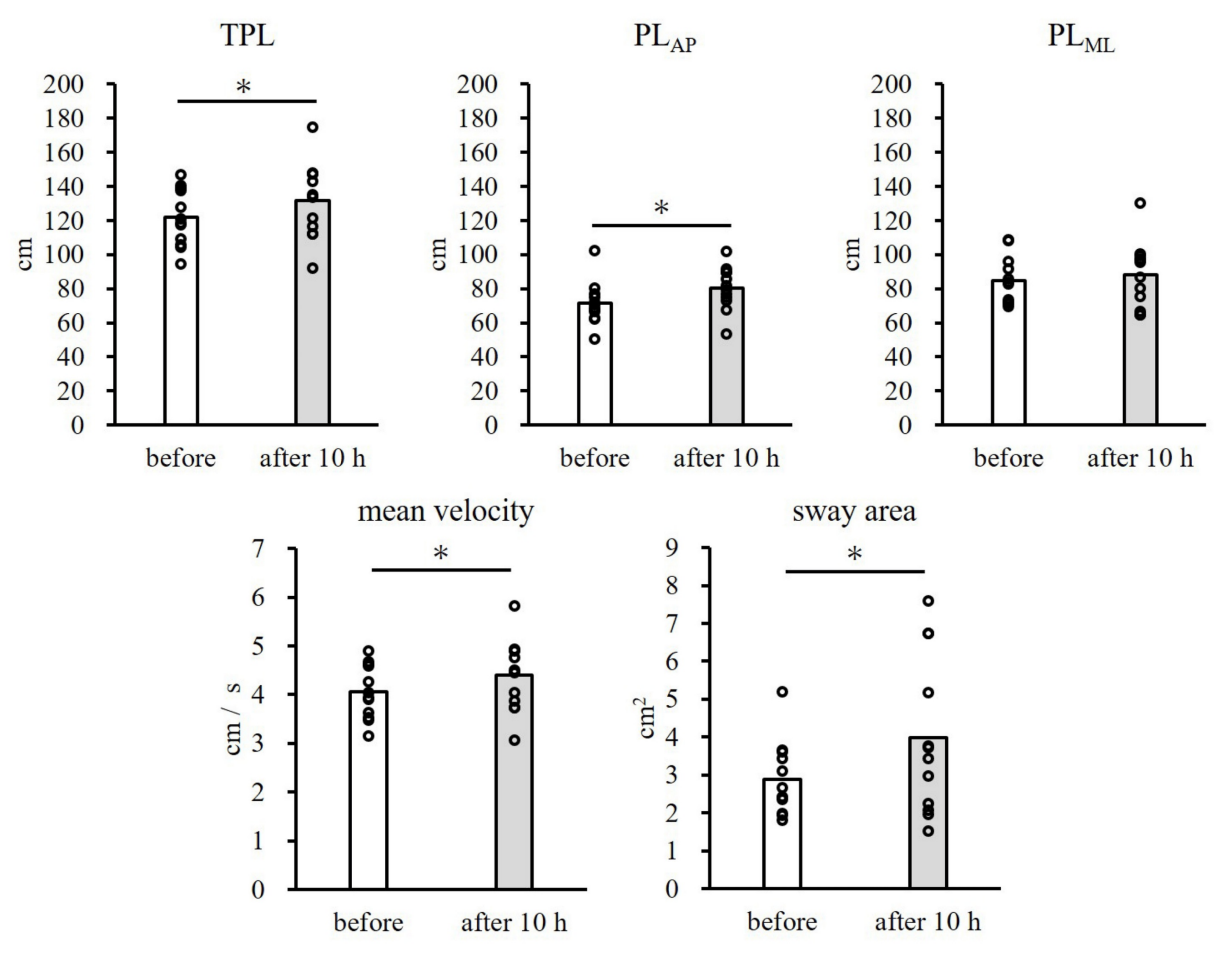
Mean ± standard deviation of the COP movements under conditions before and after 10 hours. COP: center of pressure; TPL: total path length; PL_AP_: anterior-posterior path length; PL_ML_: medial-lateral path length *: Significant difference (p < 0.05)

No participants dropped out during this study. The changes in COP before and after cast removal are summarized in Figure 1. The TPL significantly increased from 121.9 ± 16.0 cm to 131 ± 21.3 cm after cast removal compared to before cast application (p = 0.04, d = 0.54). Similarly, the PL_AP_ significantly increased from 71.3 ± 11.9 cm to 80.5 ± 12.2 cm after cast removal (p < 0.01, d = 0.76), whereas the PL_ML_ demonstrated no significant changes after cast removal (84.6 ± 13.4 cm before vs. 88.2 ± 18.4 cm after, p = 0.40, d = 0.23). Mean velocity and sway area increased after cast removal compared with values before cast application (mean velocity: 4.1 ± 0.5 cm/s before vs. 4.4 ± 0.7 cm/s after, p = 0.04, d = 0.54; sway area: 2.9 ± 0.9 cm^2^ before vs. 4.0 ± 2.0 cm^2^ after, p = 0.03, d = 0.71).

**Figure 2:**
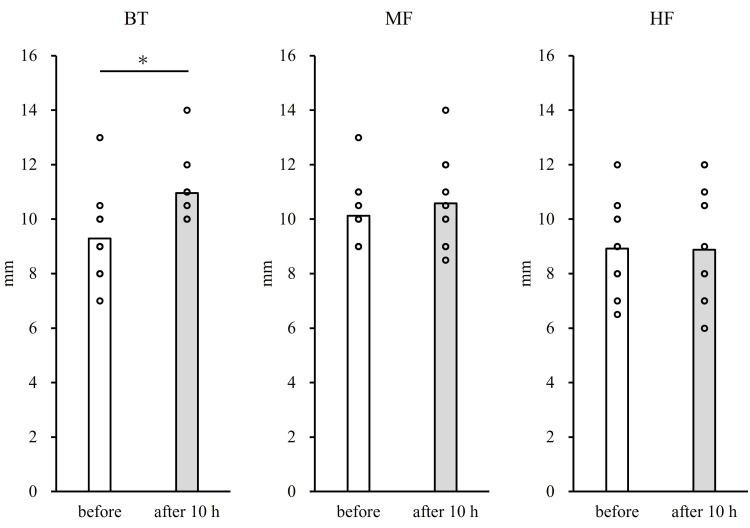
Mean ± standard deviation of the two-point discrimination sensation distance under conditions before and after 10 hours. BT: big toe; MF: mid foot; HF: heel foot *: Significant difference (p < 0.05)

The TPD sensation distances before and after 10 hours are summarized in Figure 2. TPD at the BT significantly increased from 9.3 ± 1.5 mm to 11.0 ± 1.1 mm after cast removal, whereas the MF and HF demonstrated no significant changes after cast removal (BT: p < 0.01, d = 1.29; MF: 10.1 ± 1.1 mm before vs. 10.6 ± 1.6 mm after, p = 0.20, d = 0.36; HF: 8.9 ± 1.5 mm before vs. 8.9 ± 1.9 mm after, p = 0.94, d = 0.02).

**Figure 3:**
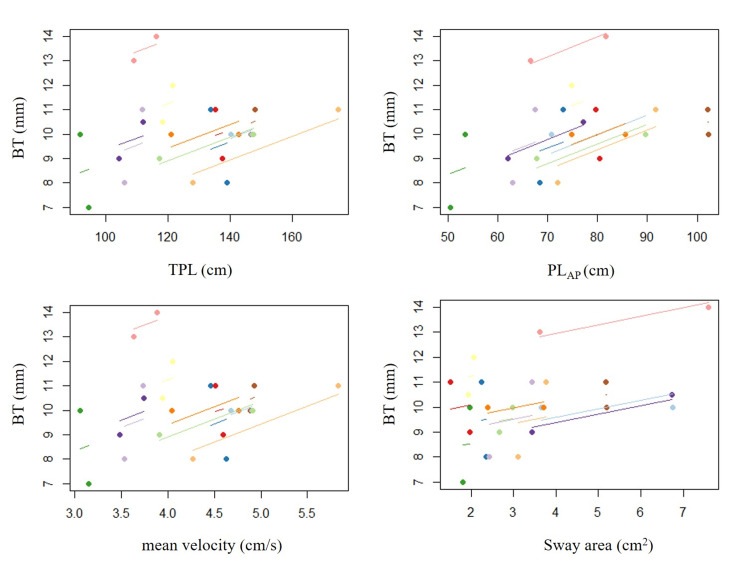
Scatter plot with repeated measures correlation Scatter plot with repeated measures correlation analysis (RMC) showing the relationship between the amount of COP movements and big toe sensation before and after the 10-hour immobilization period. Each dot represents the number of COP movements and big toe sensation, each color identifies a participant, and colored lines show the RMC fit for each participant. TPL: total path length; PL_AP_: anterior-posterior path length; BT: big toe; COP: center of pressure

A scatter plot of the RMC is shown in Figure 3. No significant correlations were found between the BT and TPL, PL_AP_, mean velocity, and sway area (TPL, p = 0.14, r = 0.44; PL_AP_, p = 0.08, r = 0.51; mean velocity, p = 0.14, r = 0.44; sway area, p = 0.30, r = 0.31).

## Discussion

This study investigated the changes in and the relationship between postural sway during one-leg standing and foot sensation after 10 hours of unilateral lower limb immobilization. To the best of our knowledge, this is the first study to examine the relationship between postural stability and sensory changes after short-term movement restriction. The results showed that COP movements increased, whereas BT sensitivity decreased after cast removal compared to before cast application. Nevertheless, no significant correlation was observed between these factors. Because these significant differences were observed in healthy adults, our findings suggest that changes in COP movements and foot sensations are acute plasticity changes caused by movement restrictions. However, COP movements were not associated with foot sensation; hence, another factor may have caused the sway after cast removal. In our experiment, we found that postural instability after cast removal may be maintained by an exploratory strategy involving multiple sensory sources to keep the body in equilibrium.

The first major observation was that TPL, PL_AP_, mean velocity, and sway area all increased after cast removal compared to before the cast application. Previous studies on short-term disuse have utilized transcranial magnetic stimulation to investigate neurophysiological changes in descending pathways from the primary motor cortex. These studies showed that unilateral limb immobilization for only 8-12 hours reduced the amplitude of motor-evoked potentials, suggesting that short-term physical inactivity may trigger synaptic depression in the motor cortex of healthy adults [10,16-18]. The frontal and parietal lobes are activated when the balance is unstable [19]. The cerebral cortex, including these regions, may regulate the excitability of subcortical postural centers to maintain balance [20,21]. Thus, short-term disuse may have caused synaptic depression in the motor cortex and inhibited neural networks in the brain from maintaining posture. Notably, the PL_ML_ showed no significant changes after cast removal. This could be because the base of support in the M-L direction is narrower during one-leg standing compared to a bipedal position, suggesting that postural control in this direction might be less affected and compensated for in the A-P direction.

The second major observation was that BT sensitivity decreased after cast removal because of physical inactivity. Huber et al. reported that immobilization of the upper limb for 12 hours reduced the amplitude of the P45 component of the somatosensory-evoked potential and delayed latency [10]. Thus, the processing of somatosensory information in the brain after lower limb movement restriction may modulate inhibition, as in the upper limb. The sensory unit called the sensory afferent nerve, comprises sensory nerves and mechanoreceptors that contribute to converting and transmitting sensory information to the central nervous system. In the foot sole, the density and distribution of receptive fields of fast-adapting and slowly adapting type I afferents are characterized by their increased presence in the BT [22]. Investigations of foot sole sensitivity in astronauts after space flights have shown that SA-type receptors are reduced during BT [23]. Thus, reduced sensory input from the ground may specifically affect the sensory afferent nerves in the BT, reducing their sensitivity. However, foot sole sensation is reduced by ischemia because of the compression of the lower extremity blood vessels [24]. Therefore, further experiments are required to elucidate whether our results are due to movement restriction or compression with cast immobilization.

In the upright stance, the foot sole sensation provides critical feedback to the central nervous system. The rationale is that enhanced somatosensory feedback from the feet reduces postural sway and decreases alpha event-related desynchronization activity on electroencephalogram [25]. When the TPD sensation in the foot sole is reduced, balance becomes unstable [26-28]. However, our results revealed no correlation between BT sensation and COP movement after 10 hours of movement restriction. Therefore, the COP sway after cast removal could be due to factors other than foot sensation. A previous study suggested that increased standing postural sway reflects the exploratory role of the central nervous system [29]. In our experiment, increased COP movement due to disuse may have been an exploratory strategy for several sensory sources to keep the body in equilibrium against unreliable somatosensory input.

Nevertheless, this study had a few limitations. First, the relatively small sample size may have influenced the results, thereby limiting their generalizability. Second, because the evaluations were conducted at different times on the same day, the potential confounding effect of diurnal variation cannot be ruled out. Third, we did not record brain activity, peripheral sensory afferent nerves, or peripheral circulation changes associated with movement restriction; thus, the mechanisms underlying these responses to changes in COP sway and BT sensitivity remain unknown. Further studies are required to verify our findings while addressing these limitations.

## Conclusions

The findings of this study showed that COP movements increased and foot sensitivity decreased as acute responses to cast immobilization of the unilateral lower extremity, supporting our hypothesis. However, no significant association was observed between these factors. Future studies should investigate the underlying mechanisms of static postural control after movement restriction to better understand the effects of postural dysregulation after conservative treatment with casts in lower extremity trauma cases.
